# Being sensitive in their own way: parental ethnotheories of caregiver sensitivity and child emotion regulation across five countries

**DOI:** 10.3389/fpsyg.2023.1283748

**Published:** 2023-12-22

**Authors:** Ju-Hyun Song, Sook In Cho, Gisela Trommsdorff, Pamela Cole, Shanta Niraula, Ramesh Mishra

**Affiliations:** ^1^Department of Child and Family Studies, Yonsei University, Seoul, Republic of Korea; ^2^Korea Institute of Child Care and Education, Seoul, Republic of Korea; ^3^Department of Psychology, University of Konstanz, Konstanz, Germany; ^4^Department of Psychology, Pennsylvania State University, University Park, PA, United States; ^5^Central Department of Psychology, Tribhuvan University, Kirtipur, Nepal; ^6^Department of Psychology, Banaras Hindu University, Varanasi, India

**Keywords:** culture, developmental niche, parental ethnotheories, caregiver sensitivity, emotion regulation, emotion socialization

## Abstract

Caregiver sensitivity builds a basis for children’s sense of security and effective emotion regulation during their development. Applying a cross-cultural lens, caregiver sensitivity can be divided into two subtypes, reactive and proactive, and its prevalence and meaning may differ across cultures. Guided by the theoretical frameworks of developmental niche and parental ethnotheories, the current study examines culture-specific meanings of caregiver sensitivity across five countries: India, Nepal, Korea, the United States of America (USA), and Germany. We examine the prevalence of maternal reactive and proactive sensitivity, children’s emotional lability and regulation, and how mothers’ sensitivity types are related to children’s emotional characteristics. Participants included 472 mothers from the five countries with children aged between 6 and 7 years. Mothers reported their sensitivity preference in multiple vignettes and completed an emotion regulation checklist to report their children’s emotional lability and regulation. A set of analyses of covariance (ANCOVAs) found cultural differences in mothers’ preference for proactive and reactive sensitivity. Mothers in India and Nepal reported the highest preference for proactive sensitivity followed by Korea and the USA, while German mothers reported the lowest preference for proactive sensitivity. Consequent regression analyses revealed varying associations between proactive sensitivity and child emotional characteristics in all five countries either directly or as moderated by child sex. These results evidence that parental ethnotheories are part of the developmental niche embedded in a larger cultural context. Findings on the differential links between the types of sensitivity and child emotion regulation provide cultural models of parental emotion socialization and children’s emotional functioning.

## Introduction

Cultural norms, values, and practices influence how individuals interact with one another and structure their social relationships (Whiting, 1980). This concept underpins the “developmental niche” framework, which posits that children are embedded within three highly interrelated, culturally constructed environmental subsystems (Harkness and Super, 2020). These subsystems consist of physical and social settings (e.g., school, household), childcare customs (e.g., age limit to be considered a dependent, level of involvement in the child’s schooling), and the caregivers’ personal beliefs (e.g., concerning maturational milestones, obedience).

All these aspects of a developmental niche have been found to interact with child-level characteristics, such as sex and age, as well as with different levels of the broader environments surrounding the child and caregiver, including that region’s dominant cultural values (Levine, 1974; Whiting, 1980; Bronfenbrenner and Morris, 2007). Among the three subsystems of the developmental niche, caregiver beliefs have been found to be particularly sensitive to and influenced by the society’s dominant cultural values. This influence on caregiver beliefs can then, in turn, have consequential effects on other aspects of the child’s developmental niche, including customs for childcare or the makeup of the child’s social settings. Parental beliefs regarding the appropriate direction of socialization for children, stemming from one’s culture-specific understandings of and views on proper parenting practices along with what is considered to be “ideal” child development, have also been referred to and further conceptualized as *parental ethnotheories* (Harkness and Super, 2020). Parents holding autonomous parenting ethnotheories emphasize the development of early childhood self-regulation and a contingent (versus a more preemptive or proactive) reaction to their children’s signals, while parents holding a more relational form of parental ethnotheory place greater emphasis on intimate parent–child interactions and a more immediate or proactive satisfaction of their child’s needs (Gartstein and Putnam, 2019).

Although an increasing number of studies on parental ethnotheories and socialization practices have focused on culturally diverse groups, there are still several limitations in this area. First, the potential meanings of seemingly identical parental socialization beliefs, measured with tools typically developed in Western societies, have not been interpreted within a larger cultural context. Second, with some exceptions (e.g., McCord and Raval, 2016; Cho et al., 2022), the implications of certain parental ethnotheories and socialization practices for children’s developmental outcomes are often assumed to be universally positive or negative, an assumption which still needs more studies conducted across (and within; see next limitation) diverse cultural groups to support. Third, studies that examine between-cultural comparisons rarely also consider within-culture variations. The effects on parental socialization practices and child developmental outcomes of within-culture, individual level factors, such as socioeconomic status, parent age, or child gender (see also Deater-Deckard et al., 2018) have yet to be thoroughly studied in conjunction with and compared to the effects of between-culture variation in a single multinational study.

The current study aims to address these gaps by using the framework of developmental niche to examine the meanings of a specific parental socialization belief. Specifically, we focus on caregiver sensitivity, which has been considered important not only for establishing secure attachment in infancy, but also for various adjustment outcomes in childhood (Ainsworth et al., 1978; Khaleque, 2013). However, sensitivity, a fundamental concept in attachment literature, has been observed to demonstrate a bias towards Western cultural perspectives (Morelli et al., 2017; Keller et al., 2018). In the current study, we compare the prevalence of parental endorsement of different forms of caregiver sensitivity and the relationships between these different forms and child emotion regulation across different five countries that vary culturally.

### Caregiver sensitivity within a developmental niche

*Caregiver sensitivity* can be defined as a caregiver’s ability to respond to children’s needs promptly and appropriately (Trommsdorff and Friedlmeier, 2010). It has been regarded a critical social factor that builds a basis for children’s sense of security and social–emotional competence (Ainsworth et al., 1978; Grusec and Davidov, 2010). Caregiver sensitivity is considered an essential component of positive parenting and has been associated with adaptive outcomes for children: children’s ability to build secure attachments (Antonucci and Levitt, 1984; Seifer et al., 1996), to regulate their emotions (Thompson et al., 2013; Frick et al., 2018), to achieve positive social adjustment (Song et al., 2019) and cognitive competence (Feldman et al., 2004). Most developmental studies on caregiver sensitivity, however, have conceived of sensitivity as a universally valid construct, largely ignoring cultural specifics regarding the expression and function of caregiver sensitivity.

A debate on the universality and cultural specificity of caregiver sensitivity is going on especially in the attachment literature. Mesman et al. (2018) argued that the function of sensitivity is universal while its manifestation is not uniform across cultures. For example, infants are soothed mainly through physical means with little verbal communication in non-Western communities (e.g., South Africa, East Asia) whereas the Western patterns (e.g., North America, Europe) rather include verbal and face-to-face interactions. According to Ainsworth et al.’s (1978) definition of caregiver sensitivity, both cultural practices can be viewed as functionally sensitive: responding contingently to the child’s signals and communications.

Meanwhile, Keller et al. (2018) argue that sensitive responsiveness as defined by attachment theorists is not universal, and thus not only its format but also its function varies across cultural contexts. For example, sensitive parents in Western countries let their child take the lead in interactions by responding to the child’s expression of needs while focusing on the child as the central social agent. In contrast, non-Western parents keep their child in proximity, where self–other boundaries are blurred, parents speak on behalf of children, and children learn to attend to the social demands of the group. Further, a main criticism regarding implications of sensitivity points out to the cultural specificity of mother–child bonds and interactions. For example, in the Global South, including African and Latin America, infants and children are growing up in contexts of multiple parenting, where children are cared for by a network of individuals beyond their biological parents including relatives and neighbors (Morelli et al., 2017). Accordingly, the evaluation of what is prompt and appropriate in responding to children’s needs may depend on specific cultural values and customs. In other words, some cultures may prioritize teaching children to be independent from a young age, while others may emphasize group harmony and cooperation through responding to a child’s needs.

The present study attempts to examine the cultural meanings of sensitivity in childhood. Our focus is on the question what mothers in different cultural communities consider contingent responses. Keller et al.’s (2018) idea on cultural differences in caregiver sensitivity across various developmental niches is in line with Rothbaum et al. (2006) study on preschool teachers. They have shown the theoretical and empirical importance to distinguish between proactive and reactive forms of sensitivity. Their results illustrate a basic difference between Japanese and American socialization practices: *anticipating* a child’s frustrations and acting to minimize self-focused negative emotions versus *reacting* to a child’s negative emotions and fostering the child’s self-reliance and independence. The authors have developed a valid method to measure the different meaning of sensitivity cross-culturally. Sensitivity is defined as reactive if caregivers contingently respond or react to children’s expressed needs; sensitivity is defined as proactive if caregivers promptly satisfy children’s anticipated needs before these needs are expressed (Rothbaum et al., 2006).

Although limited, previous cross-cultural research on parental socialization has also suggested that the prevalence and outcomes of proactive and reactive sensitivity may differ across countries and reflect culturally nuanced values and beliefs (e.g., Ziehm et al., 2013; Cheah et al., 2018; Vu et al., 2018). Caregiver sensitivity has been characterized as more reactive than proactive regarding children’s expressions of needs in Western societies (e.g., Germany), as a main socialization goal of caregivers in these societies is to foster a sense of independence and self-assertion. In cultures that encourage self-expression and emotional expressiveness, caregivers socialize their children to express their needs and emotions openly, and responding to them contingently is a characteristic of sensitive caregivers (Trommsdorff and Friedlmeier, 2010; Park et al., 2012). In non-Western societies (e.g., Japan), however, sensitivity has been reported to function as proactive behavior, since socialization goals emphasize attunement to and empathic relationships with others in line with the key values of interdependence (Rothbaum et al., 2006). Also, the open expression of needs is not considered socially desirable while the suppression of emotions is encouraged (Ramzan and Amjad, 2017); thus, parents may try to anticipate their children’s needs by interpreting subtle situational cues (Park et al., 2012). In sum, proactive sensitivity may be more preferred by parents in countries where group harmony and intimate interpersonal relationships are prioritized whereas reactive sensitivity may be more commonly endorsed in countries where self-expression and independence are highly emphasized. Understanding the prevalence of a parenting practice is a way to understand its cultural normativeness, which functions as a moderator of certain associations between parenting practices and child outcomes (Lansford, 2022). Taken all together, it is meaningful to study whether different parental ethnotheories of caregiver sensitivity exist and how they may structure the environment of children’s emotion socialization.

### Parental ethnotheories and child emotional outcomes across cultures

Efforts to examine parental ethnotheories are aiming to contribute to a culturally sensitive conceptualization of socialization practices (e.g., love withdrawal, corporal punishment) and their effects on child development across different developmental niches (Harkness and Super, 2000; Vu et al., 2018). For example, mothers’ unsupportive reactions to children’s negative emotions are associated with children’s emotional development differently across cultures; European and American mothers’ dampening reactions to negative emotions are related to a lower level of child emotional knowledge while this is not the case in Chinese American families (Song et al., 2019). Unsupportive maternal reactions to children’s negative emotions are positively related to behavioral problems in children in European and American families but not in children from Indian immigrant families (McCord and Raval, 2016). These suggest that seemingly similar parenting practices may have different meanings and functions that are related to respective cultural contexts and related value orientations and agency beliefs.

Studying caregiver sensitivity in diverse cultural contexts has thus far provided inconsistent and limited evidence concerning its implications for child development. Using Q-sort, Ekmekci et al. (2015) compared the level of caregiver sensitivity across different countries and found evidence for cross-cultural similarities. Meanwhile, Posada et al.’s (2016) results on the differences in the relationship between caregiver sensitivity and attachment security underline the importance of considering the domain specificities of sensitive behaviors. However, parental ethnotheories of proactive and reactive sensitivity have not been directly compared across cultures to understand their respective prevalence and consequences in different developmental niches. Our study’s unique focus addresses cross-cultural comparisons of the contingency of maternal responses to children’s needs and their relation to child emotional development beyond infancy.

### Emotion regulation across cultures

One approach to understanding emotional development is to examine emotion regulation capacity, a key developmental task in childhood (Trommsdorff and Cole, 2011). Effective emotion regulation is characterized by the ability to calm negative emotions, understand one’s own and others’ emotional states, and express emotions in a socially appropriate manner, including strategies to both control negative emotional arousal and engage in positive social interactions (Shields and Cicchetti, 1997). From a culturally informed perspective, emotion regulation also means adaptation to cultural mandates and prominent values such as prioritizing interpersonal relationships (Trommsdorff and Kornadt, 2003). According to De Leersnyder et al. (2013) emotion regulation can be regarded as *cultural regulation*—a cultural and developmental process involving the alignment of emotions with the values, goals, and concerns of each culture. This process is key to understanding how emotional experiences tend to be congruent with cultural values and how emotion regulation takes place at both individual and relational co-regulation levels. In other words, an individual’s need to maintain good relationships with other members of society may motivate their emotion regulation (Gross et al., 2006).

Some empirical evidence supports cultural regulation shaping individuals’ culturally adaptive emotion regulation strategies. For example, Schunk et al. (2022) found that ruminating and suppressing negative emotions were more commonly reported strategies and less correlated with mental health in Japanese than in German college students. Moreover, the positive link between suppressing negative emotions and mental health was mediated through an interdependent self-construal among Japanese but not German students, indicating culture’s moderating effect in the association between emotion regulation strategies and mental health indices.

Parents also encourage certain emotional experiences more than others by shaping a child’s proximal environment within a developmental niche as they co-regulate the child’s emotional experiences (De Leersnyder et al., 2013; Harkness and Super, 2020). Limited evidence has suggested that parents in independence-promoting cultures use more autonomy-granting and less directive parental strategies (Rothbaum and Wang, 2010; Corapci et al., 2018). However, competent self-regulation, including emotion regulation and delaying gratification in early childhood, was predicted by parents’ interdependence orientation and responsive parenting control (Lamm et al., 2018).

Despite inconsistent findings on the relationship between cultural orientation and children’s self-regulation (Jaramillo et al., 2017), parents’ perceptions of children’s emotion regulation and strategies for fostering children’s emotion regulation may systematically vary across cultures based on culture-specific ethnotheories (Trommsdorff et al., 2012; Benga et al., 2019; Harkness and Super, 2020). In cultures that strongly emphasize intimate mother–child relationships and exerting self-regulation according to social norms, children are perhaps more likely to be perceived as needing parental help and are viewed as emotionally immature. In cultures where independence and self-expression are emphasized, on the other hand, children may be viewed as emotionally mature and independent (Gartstein and Putnam, 2019).

### Maternal sensitivity and child gender

Parental ethnotheories are not only influenced by dominant cultural values in a society but also by societal beliefs regarding gender (Whiting, 1980; Wood and Eagly, 2012). Gender socialization literature has shown that the meaning of gender is culturally sculpted, and parents tend to expect more independence and autonomy for boys and more interpersonal sensitivity and close relationships for girls based on gender-specific societal expectations. For example, parents are more likely to foster close relationships and expressiveness with daughters than with sons through more supportive and directive speech (Leaper, 2013). Additionally, the expression of the internalizing affect (e.g., fear, sadness) is perceived to be less masculine, and the display of emotions (e.g., anxiety) is discouraged in boys (Jansz, 2000). These results from mostly North American and European studies suggest that societal gender beliefs may discourage parents’ proactive sensitivity to boys’ needs. Given these findings, mothers endorsing higher proactive sensitivity for girls than boys may be more consistent with social norms and is thus related to adaptive emotional outcomes in children.

Although there is growing evidence of gender-differential socialization practices and changes in their patterns, their consequences regarding boys’ and girls’ development have generally been understudied (Root and Denham, 2010). Some researchers have found that differential socialization of emotions in boys and girls may contribute to different emotion expression and adjustment outcomes in girls and boys (Whiting, 1980; Zahn-Waxler et al., 2008; Chaplin and Aldao, 2013). However, the respective cultural differences are largely unknown. Additionally, societal changes have de-emphasized, or even strongly discouraged, gender-differentiated roles in postindustrial societies since gender attitudes became more egalitarian and flexible during the last few decades of the 20th century (Leaper and Farkas, 2015). Accordingly, gender differences regarding the effects of maternal sensitivity may be more salient in traditional cultures that adhere more closely to strict differentiation between gender roles (Marshall, 2016; Lansford, 2022). The lack of sufficient evidence and mixed results call for an examination of gender differences in mothers’ use of sensitivity and the moderating effects of gender on child emotional outcomes across different developmental niches. In the present study, we examine intracultural variations as a function of child sex, considering socialization effects at the intersection of child gender and culture.

### The present study

This study’s overarching goal is to explore specificities in the forms and effectiveness of sensitivity across developmental niches. We compared mothers’ parental ethnotheories regarding proactive and reactive sensitivity and perceptions of children’s emotion regulation for investigating their associations across culture and child sex. First, we examined mothers’ endorsements of proactive vs. reactive sensitivity across five countries, India, Nepal, Korea, Germany, and the USA. We hypothesized that mothers’ ethnotheories of proactive sensitivity follow their culture’s pattern of interdependence: the more a culture promotes group harmony and intimate relationships, the more mothers exhibit proactive sensitivity. Therefore, we expected that India and Nepal, which traditionally value group harmony, would exhibit a stronger preference for proactive sensitivity, while Germany and the USA would demonstrate higher levels of reactive sensitivity due to their emphasis on self-expression and independence.

Second, we examined mothers’ perceptions of their children’s positive and negative aspects of emotion regulation across five countries. Based on the literature on cultural differences in parental expectations for self-regulation (Chen et al., 2019; Gartstein and Putnam, 2019), we hypothesized that mothers in Nepal and India perceive poorer emotion regulation, whereas mothers in the USA and Germany perceive better emotion regulation in their children.

Third, we tested the relationships between the endorsement of proactive and reactive sensitivity and child emotion regulation across five countries. Relying on the notion that the concordance between parental socialization practices and specific cultural norms is related to children’s adaptive functioning (McCord and Raval, 2016; Song et al., 2019), we expected that proactive sensitivity would be more positively associated with certain emotional outcomes (i.e., higher positive regulation, lower negative lability) in countries that emphasize more group harmony and relational intimacy (i.e., India, Nepal, Korea) than autonomy and self-expression (i.e., the USA, Germany).

Finally, we explored the moderating role of child sex in testing the association between sensitivity and child emotion regulation. In accordance with the gender socialization literature, we expected mothers’ proactive sensitivity to be more strongly related to emotion regulation in girls than in boys. Additionally, building on previous evidence supporting more pronounced gender differences in emotion socialization in traditional societies (e.g., Pierrehumbert et al., 2009; Marshall, 2016), we expected to find stronger sex moderation effects in Nepal and India relative to the other three countries examined in our study.

## Methods

### Participants

The participants were mothers of 472 children across five countries: 89 Indian, 99 Nepali, 100 Korean, 83 American, and 101 German mothers of children who were attending the first grade in primary school and were between 6 and 7 years of age. We focused on the developmental transition period of first grade because it is less culturally idiosyncratic than experiences in kindergartens or preschools. The research team leader of each country recruited the participants through convenient sampling, including kindergartens, schools, registration offices, and email listservs.

Priority was placed on recruiting mothers embedded in similar cultural contexts for socialization and parenting practices who share in similar social expectations surrounding parental success and the successful socio-emotional development of their children, resulting in our selection of mothers from each of the five countries based on shared regional locale (i.e., similar physical, socioeconomic, socio-political, and cultural environments). While considering how our findings may generalize to the regional populace level, we do cautiously infer how our findings would apply more broadly to cross-cultural comparisons at the country level as well.

Nepali mothers were from Kathmandu and belonged to the Brahmin or Chhetri ethnic group, emphasizing strong social relations. The Brahman, high Hindu Nepalese caste, followed by Chhetri, played a dominant role in shaping modern Nepalese culture, occupying a significant position. These two castes together constitute the largest group in Nepal, sharing customs that emphasize discipline and obedience. Indian mothers were recruited from Varanasi, an ancient city of Hindu tradition and culture in which human social values such as tolerance, sharing, compassion, nurturance of the young and obedience to elders are greatly emphasized (Tripathi, 1988; Mishra, 2012). Along with many social and cultural changes taking place in the Indian society, these values have been coexisting with individualistic values such as emphasizing personal happiness and economic gains (Sinha and Tripathi, 1994). These two South Asian countries share cultural similarities rooted in oriental traditions, emphasizing family values, humility, and obedience (Schwartz, 2006; Government of Nepal, n.d.). Korean mothers, who are racially and ethnically homogeneous, were recruited from Seoul, a highly developed metropolitan city. Mothers from Seoul prioritize cultural values of respecting elders and hierarchy, as well as group harmony, while placing high emphasis on education and academic achievements (Kim et al., 2005). The sample from the USA primarily consisted of white mothers living in either the college town or the surrounding rural/semi-rural communities in Pennsylvania. Employment in the region mainly comes from a large university, farming, limited small industries, and independent businesses, with most families identifying as Christian and having both conservative and liberal orientations. German mothers were recruited from Konstanz, a middle-sized university city with a mix of industry, tourism, and small enterprises. German mothers exhibit high civic engagement (e.g., solidarity; joint preparation of festivals) and relatively high levels of well-being and life satisfaction (e.g., Findeisen et al., 2009). In general, Western Europe and the USA share similar cultural emphasis on autonomy, social responsibility, and personal freedom (Hofstede Insights, n.d.), but the priority of values endorsed in Germany leans towards mastery, harmony, egalitarianism, and intellectual autonomy, while the USA emphasizes mastery, hierarchy, and embeddedness more (Schwartz, 2006).

Socio-demographic information for the five samples is detailed in Table 1. Comparative analyses revealed that the samples were equivalent in terms of economic status because samples from five countries were generally middle class from urban areas; however, there were significant group differences in mothers’ age, education, work status, and the number of children in the household. American and German mothers were older than Korean mothers, and Korean mothers were older than Indian and Nepalese mothers. Mothers’ education levels were the highest in Indian mothers (all received above-high school degrees), followed by American and Korean mothers, and Nepali (more than 40% received high school degree or below) and German (⅓ received high school degree or below) mothers had relatively the lowest education. Also, more American and German mothers were working compared to Indian, Nepalese, and Korean mothers. Finally, American and German mothers had more children than Indian, Nepalese, and Korean mothers. These socio-demographic factors were therefore controlled for in the main analyses to examine the cross-national cultural differences independent from the demographic similarities across the five sample groups.

**Table 1:** Socio-demographic characteristics of the participants across five countries.

	India (*n* = 89)	Nepal (*n* = 99)	Korea (*n* = 100)	United States (*n* = 83)	Germany (*n* = 101)
Mother					
Age^a^	33.02 (4.04)_a_	31.69 (4.09)_a_	36.23 (3.24)_b_	38.80 (5.37)_c_	40.43 (4.47)_c_
Education^b^	0/89_a_	44/55_b_	14/86_c_	7/76_a, c_	31/70_b_
Work status^c^	71/18_a_	67/32_a_	67/33_a_	25/58_b_	25/76_b_
Economic status^d^	3.03(0.32)_a_	2.98(0.57)_a_	3.07(0.74)_a_	3.07(0.74)_a_	3.16(0.66)_a_
# of children^e^	1.94(0.71)_a_	1.82(0.56)_a_	1.94(0.53)_a_	2.68 (1.20)_b_	2.34 (1.05)_b_
Child					
Sex^f^	45/44_a_	49/50_a_	55/45_a_	45/35_a_	55/46_a_

*M*(SD) reported for mother and child age, economic status, and # of children. Frequencies reported for work status, economic status, and child sex. For education (high school degree and below/above), working status (not working/working), and child sex (boy/girl), χ^2^ difference test was performed. For others, ANOVAs were followed by *post hoc* tests with Games-Howell (equal variance unassumed, unequal sample size); different subscripts within a row were significantly different at *p* < 0.05. ^a^*F*(4, 467) = 72.63, *p* < 0.001, η^2^ = 0.38. ^b^χ^2^(4, *N* = 472) = 74.65, *p* < 0.001. ^c^χ^2^(4, *N* = 472) = 91.91, *p* < 0.001. ^d^*F*(4, 466) = 1.12, *p* = 0.35, η^2^ = 0.01. ^e^*F*(4, 466) = 16.16, *p* < 0.001, η^2^ = 0.12. ^f^χ^2^(4, *N* = 469) = 1.29, *p* = 0.86.

### Procedure

Participants were recruited at kindergartens and elementary schools, as well as through online databases and email distribution lists. Mothers who agreed to participate provided informed consent and were interviewed face to face either at home or in the research lab of the collaborating institutions depending on their preferences. Trained interviewers of the respective cultural research team read each question to the mothers and recorded mothers’ answers. All measures were translated and back translated from English into the language of each country by a native speaker of each country who was fluent in English. During this process, culturally specific meanings or unique vocabularies that led to discrepancies between the original measure and the translated measure were resolved through discussions between the translator and the research team leaders. The University Research Ethics Boards in authors’ institutions in Germany and USA provided ethical approval for the study.

### Measures

#### Maternal sensitivity interview

The Caregiver Sensitivity Interview (CSI: Rothbaum et al., 2006) was adapted to examine cultural differences in mothers’ beliefs about anticipating and responding to children’s needs (see also Park et al., 2012; Trommsdorff et al., 2012; Ziehm et al., 2013). Four of the 12 scenarios, which were originally designed to assess teachers’ preferences about responding to children’s needs in the school context, were chosen and slightly modified to reflect situations that mothers would naturally encounter. In each scenario, mothers selected one of the two response alternatives using their judgment on how a mother should behave. One alternative represented parental ethnotheories on reactive sensitivity, and the other represented proactive sensitivity [e.g., “Would you think it is important for a mother (A) to observe a child always carefully so that you know when to offer help or (B) to wait until the child requests it?”—(A) is coded as a proactive sensitivity, and (B) is coded as a reactive sensitivity]. The other three scenarios in the questions we asked were: (1) the child is playing outside and hurts him/herself; (2) the child is not feeling well and is upset; and (3) the mother’s role in meeting the child’s needs in everyday life. To determine each participant’s proportional weighing of the endorsement of proactive sensitivity versus reactive sensitivity, a proactive sensitivity score was calculated by dividing the total number of proactive sensitivity responses by the total number of caregiver sensitivity vignettes (i.e., four). Hence, reactive sensitivity has been quantified as 1 minus the proportion score of proactive sensitivity. Consequently, the reverse of a high level of reactive sensitivity is conceptualized here as a low level of proactive sensitivity.

#### Emotion regulation checklist

The Emotion Regulation Checklist (Shields and Cicchetti, 1997) was used to assess children’s emotional characteristic, which is a widely used instrument to assess children’s emotional lability, intensity, flexibility, and contextual appropriateness of expression. This measure consists of *Negative Lability* and *Positive Regulation*: Negative lability indicates greater dysregulation of (e.g., exhibits wide mood swings, is prone to angry outbursts easily, is overly exuberant when attempting to engage others in play, flat affect). Positive regulation indicates appropriateness of emotional expression (e.g., displays appropriate negative emotions in response to the acts by peers, responds quickly to friendly overtures by adults, transitions well from one activity to another). Validity of this measure has been strongly established using other observational measures of children’s regulatory abilities and expressed affect (Shields and Cicchetti, 1997). Mothers rated each item on a 4-point Likert scale (1 = *Never* to 4 = *Almost Always*), a total of 24 items. Scores for two subscales were calculated by averaging 16 items for negative lability (α = 0.73) and 8 items for positive regulation (α = 0.57). Reliability for positive regulation was modest, but this is in line with other studies using this measure as a parent-report instrument (Blandon et al., 2008; Molina et al., 2014). While translating and back-translating the questions into Hindi and Nepalese, the wording of items was slightly modified.

### Analysis plan

First, descriptive statistics were conducted to explore demographic characteristics of each sample and were compared across the five nations. Then correlational analyses were conducted to examine relations between the demographic variables and the key study variables to identify covariates for main analyses. Second, 2 (Child sex) × 5 (Country) ANCOVAs were conducted to test the differences in mothers’ preference of types of sensitivity across child sex and countries. For significant omnibus tests, Bonferroni corrections were used for *post hoc* tests to control for Type 1 error. Lastly, a set of regression analyses were conducted to examine the main effect of sensitivity and the interaction effect of child sex × sensitivity on negative lability and positive regulation across the five nations while controlling for demographic factors. For significant interactions, simple slopes were plotted for boys and girls to examine the nature of the interaction. The proportion of missing data across the study variables ranged from 0 to 4% and the result of Little’s (1988) Chi-Square Test of MCAR, χ^2^(18) = 24.18, *p* = 0.15, revealed that the data were missing completely at random, and therefore was handled using listwise deletion in SPSS version 27 (Allison, 2002).

## Results

### Preliminary analyses

Correlational analyses were conducted to examine the relations between demographic variables and the main study variables (i.e., proactive/reactive sensitivity, negative lability, positive regulation) with the total sample and within each country. When tested with the total sample, mothers’ age, *r*(453) = −0.43, *p* < 0.001, working status, *r*(453) = −0.28, *p* < 0.001, and the number of children in a household, *r*(453) = −0.16, *p* = 0.001, were negatively correlated with proactive sensitivity. Meanwhile, mothers’ age, *r*(472) = 0.19, *p* < 0.001, working status, *r*(472) = 0.20, *p* < 0.001, and family economic status, *r*(471) = 0.18, *p* < 0.001, were positively correlated with child positive regulation. On the other hand, mothers’ age, *r*(472) = −0.25, *p* < 0.001, working status, *r*(472) = −0.17, *p* < 0.001, and family economic status, *r*(471) = −0.15, *p* = 0.001, were inversely correlated with child negative lability.

When tested within each country, the number of children was positively correlated with negative lability in Indian families, *r*(89) = 0.31, *p* = 0.003. Nepalese mothers’ working status was negatively correlated with proactive sensitivity, *r*(99) = −0.24, *p* = 0.02, and education was positively correlated with negative lability, *r*(99) = 0.20, *p* = 0.05. Korean families’ economic status was correlated with positive regulation, *r*(100) = 0.29, *p* = 0.004. For American families, the number of children was negatively correlated with positive regulation, *r*(82) = −0.24, *p* = 0.03, and education level was negatively correlated with their endorsement of proactive sensitivity, *r*(70) = −0.24, *p* = 0.04. Finally, German families’ economic status was positively correlated with positive regulation, *r*(101) = 0.27, *p* = 0.01, and inversely correlated with negative lability, *r*(101) = −0.24, *p* = 0.02.

Based on the significant correlations above, mothers’ age, working status, education level, and the number of children in a household were included as covariates in the main analysis for sensitivity. Mothers’ age, working status, the number of children in a household, economic status, and education level were included as covariates in the main analyses for child negative lability. Mothers’ age, working status, family economic status, and the number of children in a household were included as covariates in the main analyses for child positive regulation.

### Endorsement of proactive/reactive sensitivity

**Figure 1:**
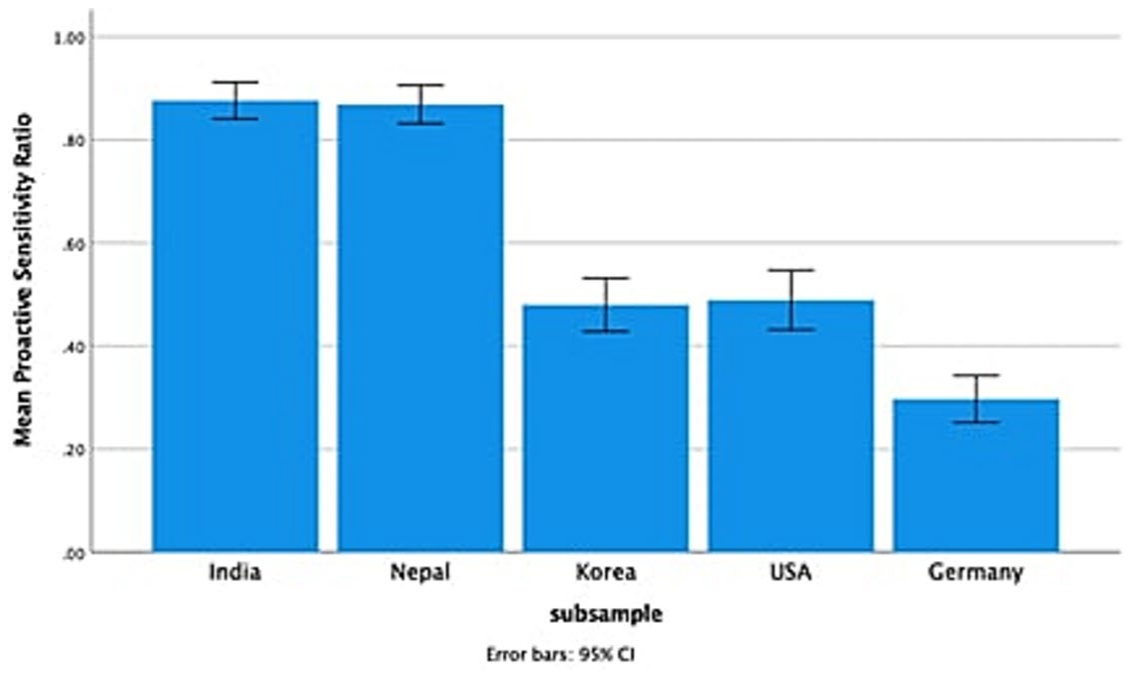
Maternal sensitivity across five countries.

As shown in Table 2, the ANCOVA conducted on sensitivity revealed a significant main effect of country, *F*(4, 437) = 76.12, *p* < 0.001, η_p_^2^ = 0.41, but no effect of child sex, *F*(1, 437) = 1.50, *p* = 0.22, η_p_^2^ = 0.003, or country x child sex interaction, *F*(4, 437) = 0.45, *p* = 0.78, η_p_^2^ = 0.004. *Post hoc* analyses showed that Nepali and Indian mothers endorsed proactive sensitivity more than Korean, American, and German mothers, and Korean and American mothers endorsed more proactive sensitivity than German mothers (Figure 1).

**Table 2:** Descriptive statistics and ANCOVA of cultural group on sensitivity, child negative lability, and positive regulation.

Mother report	India		Nepal		Korea		United States		Germany		Group differences		
	*M*	*SD*	*M*	*SD*	*M*	*SD*	*M*	*SD*	*M*	*SD*	*F*	*p*	η_p_^2^
Sensitivity	0.88_a_	0.17	0.87_a_	0.19	0.48_b_	0.26	0.49_b_	0.24	0.30_c_	0.22	76.123	<0.001	0.411
Child negative lability	2.01_b_	0.38	2.20_a_	0.35	1.80_c_	0.33	1.78_c_	0.30	1.79_c_	0.33	19.546	<0.001	0.147
Child positive regulation	3.14_d_	0.28	3.33_b, c_	0.34	3.21_c,d_	0.47	3.48_a,b_	0.35	3.53_a_	0.28	13.493	<0.001	0.106

Sensitivity scores represent the proportion of endorsement for proactive sensitivity. Different subscripts within a row were significantly different at *p* < 0.05.

None of the four covariates had a significant effect: there were no main effects of mothers’ age, *F*(1, 437) = 0.05, *p* = 0.83, η_p_^2^ = 0.00, education, *F*(1, 437) = 0.55, *p* = 0.46, η_p_^2^ = 0.001, working status, *F*(1, 437) = 0.75, *p* = 0.39, η_p_^2^ = 0.002, and the number of children, *F*(1, 437) = 0.29, *p* = 0.59, η_p_^2^ = 0.001.

### Ratings of child’s negative lability and positive regulation

As shown in Table 2, the ANCOVA conducted on negative lability revealed significant main effects of country, *F*(4, 453) = 19.55, *p* < 0.001, η_p_^2^ = 0.15, and child sex, *F*(1, 453) = 6.79, *p* = 0.01, η_p_^2^ = 0.02. *Post hoc* analyses showed Nepalese mothers reported the highest level of child negative lability than all other countries, followed by Indian mothers. Korean, American, and German mothers reported similarly lower levels of negative lability compared to Indian and Nepalese mothers. Also, mothers reported higher levels of negative lability for boys (*M* = 1.95, *SD* = 0.38) than for girls (*M* = 1.88, *SD* = 0.37), but there was no significant interaction effect of country x child sex, *F*(4, 453) = 0.47, *p* = 0.76, η_p_^2^ = 0.004.

Among the covariates, there were significant main effects of number of children, *F*(1, 453) = 4.33, *p* = 0.04, η_p_^2^ = 0.01, and economic status, *F*(1, 453) = 6.18, *p* = 0.01, η_p_^2^ = 0.01, but no effects were found for mothers’ age, *F*(1, 453) = 0.02, *p* = 0.90, η_p_^2^ = 0.00, education, *F*(1, 453) = 0.64, *p* = 0.43, η_p_^2^ = 0.001, and working status, *F*(1, 453) = 0.70, *p* = 0.40, η_p_^2^ = 0.002.

The ANCOVA conducted on child positive regulation also revealed a significant main effect of country, *F*(4, 454) = 13.49, *p* < 0.001, η_p_^2^ = 0.12, but no effect of child sex, *F*(1, 454) = 1.17, *p* = 0.28, η_p_^2^ = 0.003. Post-hoc analyses revealed Indian and Korean mothers reported the lowest level of child positive regulation. Nepalese mothers reported the middle level, and American and German mothers reported the highest level of positive regulation. There was no significant interaction effect of country × child sex, *F*(4, 454) = 0.26, *p* = 0.91, η_p_^2^ = 0.002.

Among the covariates, there was a significant main effect of economic status, *F*(1, 454) = 11.72, *p* = 0.001, η_p_^2^ = 0.03, but no significant effects of mothers’ working status, *F*(1, 454) = 0.30, *p* = 0.59, η_p_^2^ = 0.001, age, *F*(1, 454) = 0.05, *p* = 0.83, η_p_^2^ = 0.000, and the number of children in a household, *F*(1, 454) = 0.79, *p* = 0.37, η_p_^2^ = 0.002.

### Relations between caregiver sensitivity and emotion regulation

A set of hierarchical regression analyses were conducted to test the links between maternal endorsement of sensitivity and report of child negative lability and positive regulation across countries, while considering the moderating effect of child sex (Table 3). The results revealed that higher proactive sensitivity of mothers was significantly related to lower negative lability in Nepal and the US, and higher positive regulation in Korea at a trend level. No main effect of mothers’ sensitivity was found in India and Germany.

**Table 3:** Standardized regression coefficients predicting child emotion lability and regulation from mothers’ sensitivity across five countries.

Predictors	India		Nepal		Korea		United States		Germany	
	Lab	Reg	Lab	Reg	Lab	Reg	Lab	Reg	Lab	Reg
*Step1: Demo*	*R*^2^ = 0.12^*^	*R*^2^ = 0.01	*R*^2^ = 0.09	*R*^2^ = 0.03	*R*^2^ = 0.07	*R*^2^ = 0.10^†^	*R*^2^ = 0.08	*R*^2^ = 0.09	*R*^2^ = 0.09	*R*^2^ = 0.15^*^
Mother age	0.03	0.05	−0.01	0.12	−0.15	−0.02	−0.06	−0.13	−0.05	0.22^*^
Mother work status	−0.09	0.04	−0.19^†^	−0.03	0.10	0.001	0.26^†^	0.03	−0.15^†^	0.20^*^
# of children	0.28^*^	0.03	0.02	−0.13	0.05	−0.02	0.04	−0.17	0.03	0.03
Mother education^1^	–	–	0.23^*^	−0.08	−0.06	−0.14	−0.09	0.16	−0.01	0.05
Economic status	−0.11	−0.03	0.10	0.08	−0.14	0.34^**^	−0.20	−0.13	−0.26^*^	0.25^*^
*Step 2*	***Δ**R*^2^ = 0.01	***Δ**R*^2^ = 0.01	***Δ**R*^2^ = 0.09^*^	***Δ**R*^2^ = 0.03	***Δ**R*^2^ = 0.01	***Δ**R*^2^ = 0.03	***Δ**R*^2^ = 0.12^*^	***Δ**R*^2^ = 0.04	***Δ**R*^2^ = 0.05^†^	***Δ**R*^2^ = 0.05
Child sex	−0.07	0.08	−0.18^†^	0.12	−0.09	−0.01	−0.06	0.05	−0.18^†^	0.08
Sensitivity^2^	0.07	0.07	−0.25^*^	0.13	−0.04	0.18^†^	−0.35^**^	0.20	0.16	−0.05
*Step 3*	***Δ**R*^2^ = 0.001	***Δ**R*^2^ = 0.04^†^	***Δ**R*^2^ = 0.05^*^	***Δ**R*^2^ = 0.01	***Δ**R*^2^ = 0.002	***Δ**R*^2^ = 0.03^†^	***Δ**R*^2^ = 0.00	***Δ**R*^2^ = 0.01	***Δ**R*^2^ = 0.01	***Δ**R*^2^ = 0.04^*^
Sensitivity × Child sex	−0.08	0.48^†^	0.45^*^	−0.17	0.06	0.23^†^	0.02	0.12	0.20	0.35^*^

^†^*p* < 0.10, ^*^p = <0.05, ^**^p < 0.01. Child sex 0 = boy; 1 = girl. ^1^Indian mothers’ education had zero variance and was excluded from the analysis. ^2^Higher sensitivity scores represent higher endorsement of proactive sensitivity, lower sensitivity scores represent higher endorsement of reactive sensitivity.

**Figure 2:**
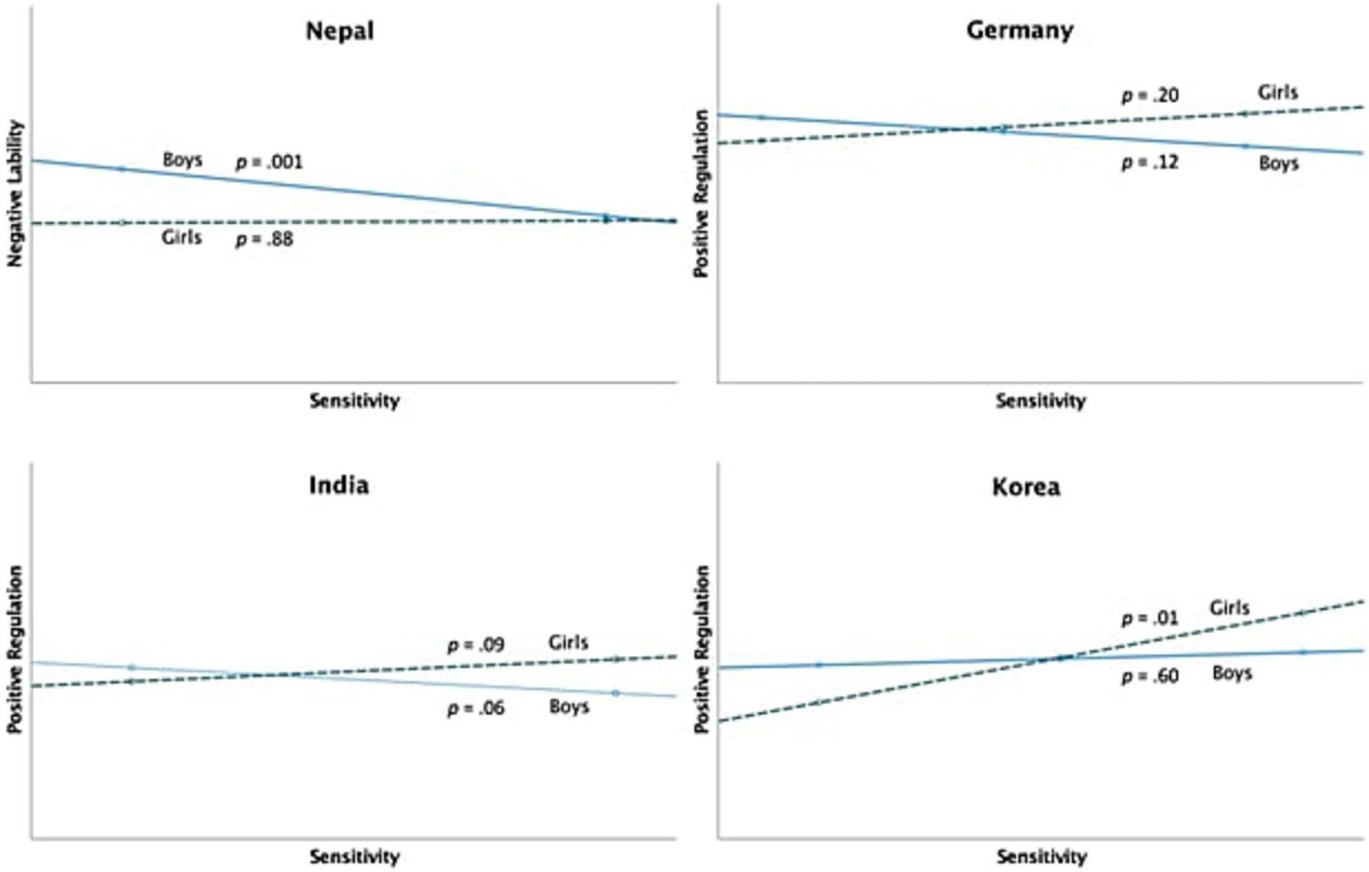
Interactions between maternal sensitivity and child emotion regulation. Sensitivity values indicate the proportion score of proactive sensitivity responses among total responses. Thus, a value to the right implies more proactive sensitivity, and a value to the left implies more reactive sensitivity.

Interaction effects between child sex and sensitivity were found in four countries except the US (Figure 2). Mothers’ sensitivity × child sex interaction was significant for children’s negative lability in Nepalese sample; when probed, reactive sensitivity was related to boys’ negative lability, *b* = −0.83, *se* = 0.23, *t* = −3.53, *p* = 0.001, LLCI = −1.29, ULCI = −0.36, whereas it was not significantly related to girls’, *b* = 0.04, *se* = 0.28, *t* = 0.15, *p* = 0.88, LLCI = −0.52, ULCI = 0.60.

The interaction was significant for positive regulation in the German sample. When probed for boys and girls separately, the association between sensitivity and positive regulation became non-significant, yet the associations were in opposite directions for boys and girls: reactive sensitivity was related to more positive regulation in boys, *b* = −0.25, *se* = 0.16, *t* = −1.58, *p* = 0.12, LLCI = −0.57, ULCI = 0.06, but proactive sensitivity was related to more positive regulation in girls *b* = 0.24, *se* = 0.18, *t* = 1.29, *p* = 0.20, LLCI = −0.13, ULCI = 0.60.

Although at a trend level, the interaction effect was found for positive regulation in India and Korea as well. In India, mothers’ reactive sensitivity was linked to more positive regulation in boys, *b* = −0.45, *se* = 0.23, *t* = −1.94, *p* = 0.06, LLCI = −0.91, ULCI = 0.01 but proactive sensitivity was related to more positive regulation in girls, *b* = 0.39, *se* = 0.23, *t* = 1.72, *p* = 0.09, LLCI = −0.06, ULCI = 0.84. In Korea, mothers’ proactive sensitivity was not related to boys’ positive regulation, *b* = 0.11, *se* = 0.21, *t* = 0.53, *p* = 0.60, LLCI = −0.31, ULCI = 0.53, but positively related to girls’ positive regulation, *b* = 0.79, *se* = 0.31, *t* = 2.58, *p* = 0.01, LLCI = 0.18, ULCI = 1.41.

## Discussion

The current study examines mothers’ parental ethnotheories of sensitivity in relation to positive and negative aspects of emotion regulation in school-age children across five countries. We also tested intracultural variations in parental ethnotheories and child emotional outcomes by examining child sex as a moderator of the associations between caregiver sensitivity and child emotion regulation. We found preliminary evidence for differences in the endorsement of proactive/reactive sensitivity across the five countries that map onto the countries’ emphasis on interdependence after controlling for demographic factors. A similar pattern was found for mothers’ perceptions of child emotion regulation; that is, children seem to be perceived as showing more negative lability and less positive regulation in Nepal, India, and Korea. The links between maternal sensitivity and child emotional outcomes showed more complicated patterns, many of which are moderated by child sex, although mothers’ proactive sensitivity was generally related to children’s high positive regulation and lower negative lability. These findings contribute to a more refined understanding of parental ethnotheories in developmental niches that are influenced by culture and child sex.

### Cultural patterns in parental ethnotheories of caregiver sensitivity

After taking into account the influence of demographic factors, there was a notable cultural pattern in mothers’ beliefs concerning proactive sensitivity: Indian and Nepali mothers endorsed proactive sensitivity the most, followed by Korean and American mothers; German mothers endorsed reactive sensitivity the most. None of the demographic factors explained the endorsement of the type of sensitivity, supporting the validity of the cultural explanation for this pattern. In Nepal and India, where group harmony and interpersonal relationship are viewed highly important, individual initiative is less prioritized (Chen et al., 2019). Thus, the contingency of responsiveness indicated by the timing of sensitivity shows varying patterns across the five countries, supporting our assumption of cultural specificities regarding the form and expression of sensitivity.

Our results support our expectation that cross-cultural variations in parental ethnotheories regarding caregiver sensitivity may be related to variations in values and beliefs that are endorsed in respective cultures (Raval and Walker, 2019). Studies that compared individuals’ culture-related attitudes and behaviors across different societies found that European and American mothers tend to focus their efforts on providing a safe environment for their children to express their emotions and beliefs and explore the environment autonomously (Vu et al., 2018). However, in traditional East Asian countries, prioritizing group harmony and interpersonal connectedness have been at the core of socialization (Markus and Kitayama, 1991; Chen et al., 2019). Parenting influenced by such “cultural mandates” tends to highlight fostering close interpersonal relationships and fulfilling family obligations (Choi et al., 2013; Vu et al., 2018).

Socialization goals of promoting group harmony and intimate relationship are consistent with the motivation underlying proactive sensitivity because of its emphasis on intimate mother–child relationship and connectedness. According to previous cultural studies, Korea is considered an autonomous-related culture, which has integrated traditional collectivistic values as well as individualistic values during its cultural transition due to industrialization and westernization (Kim et al., 2005; Kağitcibasi, 2012). Interestingly, Korean mothers in the present study showed a mixture of proactive and reactive sensitivity preferences, seemingly reflecting the mixture of different cultural values.

American mothers unexpectedly showed a level of proactive sensitivity similar to Korean mothers. This may be due to Korea’s high level of intracultural heterogeneity despite the country’s overall emphasis on self-expression and independence, in comparison to another Western society, Germany. Intracultural heterogeneity of the USA may be related to the large immigrant population. In fact, Alesina et al.’s (2003) report on ethnic, linguistic, and religious heterogeneity across 190 countries showed an overall higher fractionalization score of USA compared to Germany. In a culturally more heterogeneous society, parents may be holding more culturally diverse values. These findings underline that parental ethnotheories reflect cultural values and beliefs (Bornstein et al., 1992; Keller et al., 2006) and suggest to study variations in maternal ethnotheories of sensitivity across different cultural contexts (e.g., Rothbaum et al., 2006; Ziehm et al., 2013).

### Cultural patterns in the perceptions of child negative lability and positive regulation

The distribution pattern of mothers’ perceptions of children’s negative lability and positive regulation across the five nations is consistent with the pattern of mothers’ sensitivity. Nepali mothers perceived the highest level of negative lability, followed by Indian mothers. Korean, American, and German mothers reported lower levels of negative lability. Among the demographic factors, the number of children in the household and economic status predicted higher lability. Given that the number of children in the household was lower in India, Nepal, and Korea in our samples, we cannot conclude that the reason for the high perceptions of lability in Indian and Nepalese mothers was due to their having more children. Additionally, economic status did not differ across the countries and thus fails to explain the cultural differences in perceptions of negative lability. A similar pattern was observed for child positive regulation, except among Nepali mothers: Indian and Korean mothers’ perceptions of their children’s emotion regulation turned out to be the least positive, followed by American, Nepali, and German mothers. Family economic status, which turned out to be very similar across the countries predicted a more positive regulation. Accordingly, the cross-national differences in maternal perceptions of their children’s positive regulation cannot be explained by family economic status.

Thus, the different cultural patterns in perceptions of negative lability and positive regulation may be attributable to maternal ethnotheories that are influenced by culture. In cultures that emphasize intimate mother–child relationships, mothers may be more sensitive to their children’s emotional status and thus more prone to recognizing and being attentive to fluctuations in their children’s emotions (Chen, 2000; Mesman et al., 2016). In cultures where self-assertion and expression are emphasized, however, mothers may allow their children to resolve emotional distress independently; they are less concerned about their children’s negative lability as some psychological space is maintained between the parent and the child (Benga et al., 2019). However, it is essential to avoid oversimplifying the cultural variations, given that across various cultural contexts, particularly within high income-inequality nations, maternal caregiving often takes the form of what is commonly referred to as helicopter or intensive parenting. In this approach, mothers place a strong emphasis on safeguarding their child’s social and emotional well-being (Ennis, 2014), which appears to exhibit a notably proactive nature.

Additionally, cultural studies on socialization and child self-regulation have argued that although self-regulation is valued and encouraged in most cultures, parents in more group-oriented cultures tend to prioritize self-regulation as an innate virtue (Chao, 1995; Chen and French, 2008). On the other hand, behavioral self-regulation is often viewed as an interfering factor for children’s freedom in cultures that prioritize independence. Our results showing Nepali and Indian mothers reporting higher negative lability and Indian and Korean mothers reporting lower positive regulation in their children may be due to having higher expectation for and sensitivity to their children’s well-regulated and culturally adaptive emotion expression. These parents might be more likely to view their children as needing caregiver support in regulating their emotions and behavior. On the other hand, German and American mothers may have perceived it as an expression of autonomy and their children as more independently self-regulated.

### Cultural patterns in the link between proactive sensitivity and emotion regulation

Proactive sensitivity was related to better emotion regulation in almost all countries in either girls or boys or in both, lower negative lability in Nepal and the USA in both girls and boys, and higher positive regulation in girls in Korea and India. In Germany, a significant moderation effect of child sex was found, although the simple slopes were not significant, showing the opposing directions of influence of reactive sensitivity on boys’ higher positive regulation and girls’ lower positive regulation. These findings reveal no clear cultural divide in the effect of proactive or reactive sensitivity on child emotion regulation in opposing directions. Instead, we found some commonalities across cultures, partially supporting the universality of the function of sensitivity: proactive sensitivity is associated with positive emotion regulation capacity across all countries except for Germany; this association is qualified by child sex.

These findings do not support our original hypotheses that reactive sensitivity is associated with adaptive emotional outcomes in Germany and the USA (Western countries) and proactive sensitivity is associated with adaptive emotional outcomes in Nepal and India (Eastern countries). Proactive sensitivity might be particularly conducive to children’s positive regulation, given that positive regulation measures children’s ability to understand emotional states and use words to express emotions (Shields and Cicchetti, 1997). Proactively sensitive mothers may serve as a model for empathy and competent emotional understanding and assist children’s understanding and expression of their own emotions by asking children about their needs and feelings (Vinik et al., 2011; Drummond et al., 2014). Therefore, the way how and the degree to which mothers proactively address children’s needs may differ across cultures and child sex, but its mechanism of influencing children’s positive regulation may be similar.

### Caregiver sensitivity and children’s emotion regulation: the moderating effect of child sex

The results show that the effect of caregiver sensitivity was moderated by child sex in most countries except the USA. Specifically, proactive sensitivity seemed to be more conducive to positive regulation for girls than for boys in India, Korea, and Germany, while reactive sensitivity was related to boys’ negative lability in Nepal. This is largely consistent with our hypotheses based on culture- and gender-specific socialization effects. However, our results do not support the hypothesis that sex moderation is more pronounced in traditionally Asian countries (e.g., Nepal) compared to Western countries (e.g., Germany).

In accordance with socio-cultural expectations for males and females, parents try to foster more independence and autonomy for boys and more interpersonal sensitivity and close relationships for girls. Studies have found that parents tend to foster close relationships and expressiveness with daughters more than with sons by using more supportive speech (Leaper, 2013) while encouraging more self-assertion with sons (Leaper and Farkas, 2015). Considering the normativity of mothers’ proactive sensitivity toward girls and reactive sensitivity toward boys, it is not surprising that proactive sensitivity was more consistently related to positive regulation in girls than in boys. The other side of this result is that reactive sensitivity was related to lower positive regulation in girls. This is consistent with previous findings that girls tend to be more susceptible to unsupportive parenting than boys (Denham et al., 2010).

We also found that reactive sensitivity was related to boys’ higher negative lability but unrelated to girls’ emotion regulation in Nepal, suggesting that when mothers endorse more proactive sensitivity, their sons may be less labile. Alternatively, when boys are less labile, mothers may endorse more proactive sensitivity. The same inverse relation of proactive sensitivity and negative lability was found in the USA, without a sex moderation effect. Children who show high negative lability are characterized by high reactivity to stress and intense expression of negative emotions (Shields and Cicchetti, 1997). Therefore, their needs or emotions are readily noticed by their mothers, who do not need to engage in proactive sensitivity to anticipate their child’s emotional state. Considering the temperamental nature of negative lability, it is more reasonable to interpret that children’s negative lability might induce mothers’ reactive instead of proactive sensitivity as a part of a bidirectional and dialectic relationship between maternal socialization and child characteristics (Kuczynski et al., 2015).

In our sample, Nepal was considered the most traditional Asian country, as Indian mothers had unusually high educational backgrounds, and Korea has been widely westernized. Our significant finding for boys in Nepal is consistent with our expectation that gender differences concerning the effect of maternal sensitivity may be more salient in traditional cultures that adhere more closely to rigid gender roles. One possible mechanism is parents’ gender-specific attributions of children’s behavior. Empirical evidence related to gender-specific socialization in Asian countries is largely inconsistent; however, some studies have found that mothers tend to attribute girls’ behavior to dispositional characteristics or moral reasons while boys’ behavior is generally attributed to environmental influences or developmental factors (Gretarsson and Gelfand, 1988; Park and Cheah, 2005). Such intracultural gendered interactions warrant further replication with a larger effect size, and uncovering the specific mechanisms (e.g., gender-relevant customs, ethnotheories, physical settings) through which mothers’ sensitivity is related to child emotion regulation will benefit from utilizing qualitative methods and the framework of developmental niche in future studies.

### Limitations and future directions

As with all studies, several limitations should be noted for our study. First, although we statistically controlled for demographic factors in our analyses, this does not rule out the possibility that the cross-national differences were independent of the demographic differences across the countries. Second, we labeled each subsample’s cultural values related to nationality without directly assessing the individual endorsement of cultural values. Future studies will benefit from assessing mothers’ endorsement of cultural values to confirm its relationship to parental socialization beliefs and practices given the heterogeneity within cultures (Halberstadt and Lozada, 2011). Developing empirical measures to operationalize culture has been a challenging task in the field but remains a task for future research to move beyond cross-national or cross-ethnic comparisons and simple dichotomous categorizations (e.g., Western vs. Eastern; Trommsdorff, 2017; Krys et al., 2022; Lansford, 2022). We acknowledge that independence and interdependence values coexist in each culture, and both tendencies are part of the socialization process (Grusec and Davidov, 2010) as well as displayed by individuals contingent upon situational needs. Third, our study recruited samples from only one city from each country, which limits the generalizability of our findings to the whole population of the country. Nevertheless, the present study provides preliminary evidence for cross-national similarities and differences in maternal sensitivity and child emotion regulation; this should be further replicated with larger, representative samples in future research. Fourth, the directionality of the link between sensitivity and emotional regulation capacity could not be determined due to the study’s cross-sectional design. Most likely, the relationship between maternal sensitivity and child emotion regulation is bidirectional (Trommsdorff and Kornadt, 2003; De Leersnyder et al., 2013), but future studies can incorporate a longitudinal measurement to determine the stronger direction of influence. Finally, measures were reported by mothers only, which might have led to shared method variance bias, and observations of parenting behaviors or parent–child interactions were not conducted. Future studies should replicate the current findings using a multi-method approach to reduce the potential confounding effects of self-reporting. Moreover, using children’s self-reports or objective measures of emotion regulation are necessary to assess children’s actual emotional outcomes over and above parents’ perceptions of them, which are a part of parents’ beliefs.

Despite these limitations, the present study contributes to the culture-informed literature on caregiver sensitivity by addressing the complex issue of cultural specificity in its manifestations. Our study revealed variations in the levels of proactive or reactive sensitivity endorsed across the five countries, as well as variations in mothers’ perceptions of children’s emotion regulation, which aligned with the relative emphasis on interdependence and independence within each country. The relationship between maternal sensitivity and child emotional characteristics displayed complex patterns with many of these associations being influenced by child sex, although, in general, higher levels of proactive sensitivity were associated with children’s higher emotion regulation. The current study illuminates the cultural mechanisms underlying the link between maternal sensitivity and emotion regulation in five different countries that were chosen according to different cultural orientations. Our findings suggest the existence of diverse parental ethnotheories regarding caregiver sensitivity and their potential influence on the emotion socialization environment for children. This study also contributes to the literature on parental ethnotheories outside the cultural binary (East vs. West) by incorporating data collected from countries where only few studies have been conducted in comparison with the USA. Finally, we have examined intracultural variations as a function of child sex, considering socialization effects at the intersection of child gender and culture within a developmental niche. Future cross-cultural studies are necessary to continue to investigate variations in parental ethnotheories and the roots and implications of universal and culture-specific aspects of parental socialization and children’s emotional development.

## Data availability statement

The data analyzed in this study is subject to the following licenses/restrictions: the data that supports this study’s findings are available on request from the corresponding author. The dataset is not publicly available for privacy and ethical restrictions. Requests to access these datasets should be directed to chosook@kicce.re.kr.

## Ethics statement

The studies involving humans were approved by Research Ethics Boards at University of Konstanz and Pennsylvania State University. The studies were conducted in accordance with the local legislation and institutional requirements. Written informed consent for participation in this study was provided by the participants’ legal guardians/next of kin.

## Author contributions

J-HS: Conceptualization, Formal analysis, Funding acquisition, Writing – original draft, Writing – review & editing. SC: Conceptualization, Writing – original draft, Writing – review & editing. GT: Conceptualization, Data curation, Funding acquisition, Investigation, Methodology, Project administration, Resources, Supervision, Validation, Writing – review & editing. PC: Conceptualization, Data curation, Investigation, Methodology, Project administration, Resources, Supervision, Validation, Writing – review & editing. SN: Data curation, Investigation, Methodology, Project administration, Resources, Supervision, Validation, Writing – review & editing. RM: Data curation, Investigation, Methodology, Project administration, Resources, Supervision, Validation, Writing – review & editing.
